# Immune-based therapies for hepatocellular carcinoma

**DOI:** 10.1038/s41388-020-1249-9

**Published:** 2020-03-10

**Authors:** David J. Pinato, Nadia Guerra, Petros Fessas, Ravindhi Murphy, Takashi Mineo, Francesco A. Mauri, Sujit K. Mukherjee, Mark Thursz, Ching Ngar Wong, Rohini Sharma, Lorenza Rimassa

**Affiliations:** 1 Department of Surgery & Cancer, Imperial College London, Hammersmith Hospital, Du Cane Road, London, W120HS UK; 2 Department of Life Sciences, Imperial College London, South Kensington Campus, Exhibition Road, London, SW7 2AZ UK; 3 Tokyo Medical and Dental University, Tokyo, Japan; 4 Department of Metabolism, Digestion & Reproduction, Imperial College London, St. Mary’s Hospital, Praed Street, London, UK; 5 Medical Oncology and Haematology Unit, Humanitas Cancer Center, Humanitas Clinical and Research Center—IRCCS, Via Manzoni 56, 20089 Rozzano, Milan Italy; 6 Department of Biomedical Sciences, Humanitas University, Via Rita Levi Montalcini, 20090 Pieve Emanuele, Milan Italy

**Keywords:** Tumour immunology, Cancer immunotherapy

## Abstract

Hepatocellular carcinoma (HCC) is the third most frequent cause of cancer-related death. The immune-rich contexture of the HCC microenvironment makes this tumour an appealing target for immune-based therapies. Here, we discuss how the functional characteristics of the liver microenvironment can potentially be harnessed for the treatment of HCC. We will review the evidence supporting a therapeutic role for vaccines, cell-based therapies and immune-checkpoint inhibitors and discuss the potential for patient stratification in an attempt to overcome the series of failures that has characterised drug development in this disease area.

## Introduction

Hepatocellular carcinoma (HCC) is a prototypical inflammation-driven cancer arising on the backdrop of liver cirrhosis. Whilst the epidemiology of chronic liver disease is changing from a largely hepatitis virus B (HBV) or C (HCV) driven landscape to a predominantly metabolic one, with non-alcoholic steato-hepatitis (NASH) rapidly increasing in prevalence [1], tumour-promoting inflammation remains the common denominator that characterises the pathogenesis of HCC across aetiologies.

The high lethality of HCC, a cancer of increasing incidence [2], stems from late-stage presentation and high prevalence of concomitant liver dysfunction [3]. Curative approaches (liver resection, ablation) in early-stage HCC are affected by high recurrence rates [4] and transplantation is feasible only within rigorous oncological criteria [5]. As cancer and liver dysfunction progress, loco-regional and systemic therapy may improve patients’ survival. Whilst significant survival benefit is now achievable with optimal treatment sequencing [6], all patients within Barcelona Clinic Liver Cancer intermediate and advanced stages will inevitably die of HCC.

After decades of failures and scepticism over the potential for immune-based therapies to produce clinically meaningful disease-modulating effects, the systemic management of cancer has been recently revolutionised by the advent of immune checkpoint inhibitors (ICPI), a therapeutic class of monoclonal antibodies that can effectively induce tumour immune-rejection by targeting key co-inhibitory signals within the cancer-immunity interface. Over the past decade, anti-cancer immunotherapy with inhibitors of the programmed cell-death 1 receptor or ligand 1 (PD-1/PD-L1) and Cytotoxic T-cell antigen 4 (CTLA-4) has swiftly become standard of care across a wide range of previously untreatable malignancies including non-small cell lung cancer (NSCLC), melanoma and many others. The strong immune-mediated pathogenesis of HCC makes this tumour particularly appealing for immune-based therapies. However, the complex functional characteristics of the HCC tumour microenvironment (TME) highlight the presence of multiple non-redundant mechanisms of cancer immune-suppression, which synergise in defining a high barrier of resistance to immunotherapy. By the time HCC is diagnosed, the various functional segments of the host’s immunity are strongly geared towards immune-suppression by un-resolved pro-inflammatory stimuli that accompany liver fibrogenesis through a process now recognised as immuno-editing [7].

In the specific context of HCC, anti-tumour immune reconstitution with ICPIs has produced initial enthusiasm based on preliminary results from single-arm studies [8, 9], suggesting evidence of anti-tumour activity. With immunotherapy rapidly expanding as a novel option in the treatment landscape of HCC [10], we discuss the rationale for the development of immune-based therapies in liver cancer and review the basic immune-biologic mechanism that underlie the progression of HCC and might be exploited for therapy.

## Molecular mechanisms of hepatic immune tolerogenesis

Sitting at the functional junction between portal and arterial inflow, the liver constitutes a primary anatomical site of immune recognition, facilitated by the low pressure, low flow sinusoidal architecture capable of exposing potential pathogens to the largest reticulo-endothelial system present in the human body [11].

To protect the liver parenchyma from unopposed tissue injury, several mechanisms contribute to naturally prevent unwanted immune responses generated from exposure to microbial antigens and conserved molecular motifs known as danger- or pathogen-associated molecular patterns (DAMPs/PAMPs), making the liver a largely immune-suppressive microenvironment. The functional heterogeneity of the liver immune microenvironment is evidenced by the multifaceted nature of stromal cells including liver sinusoidal endothelial cells (LSECs), hepatic stellate cells (HSCs), liver resident macrophages or Kupffer cells (KCs) as well as numerous functional segments of the adaptive immune response including CD4+, CD8+ T-lymphocytes and NK cells [12].

LSECs are endowed with antigen-presenting capacity, being able to activate antigen specific CD4+ T-cell responses [13]. LSECs modulate immune cell recruitment through specific integrins (αLβ2, α4β1, α4β7) that facilitate capture, firm lymphocyte adhesion and subsequent chemotaxis mediated by pathways such as CXCL9-11/CXCR3, CXCL16/CXCR6 and CX_3_CL1/CX_3_CR1 [14]. In response to lipopolysaccharide (LPS), the prototypical PAMP molecule, the antigen-presentation capacity of LSECs is dampened by downregulation of constitutively expressed Major Histocompatibility Complex (MHC) class II, CD80 and CD86 molecules [15]. Key driver of this immune-tolerogenic state is the relative abundance of prostaglandin E2 (PGE2) and interleukin 10 (IL-10), two immune-suppressive mediators produced by KC and LSECs in response to chronic LPS exposure.

Transforming growth factor-β (TGF-β), another key immunosuppressive cytokine involved in liver regeneration, inflammation and fibrosis [16], is also abundant within the liver immune microenvironment and through its complex signalling and pleiotropic functional role exerts a tolerogenic effect [17].

HSCs heavily secrete TGF-β, which yields pro-fibrogenic and anti-proliferative properties [18]. Activated HSCs contribute to liver tolerogenesis by inhibiting lymphocyte infiltration, inducing PD-L1 expression and facilitate recruitment [19] and functional differentiation of T-regs when naïve CD4+ cells are recruited to professional DCs [20]. HSCs can also prevent the activation CD8+ T-cells through a CD54-mediated mechanism by reducing IL-2/IL-2R T-cell signalling [21] and facilitate the generation of myeloid derived suppressor cells (MDSC) [22] suggesting their prominence as immune-regulatory cells despite the fairly limited capacity of functioning as APCs [23].

**Fig. 1:**
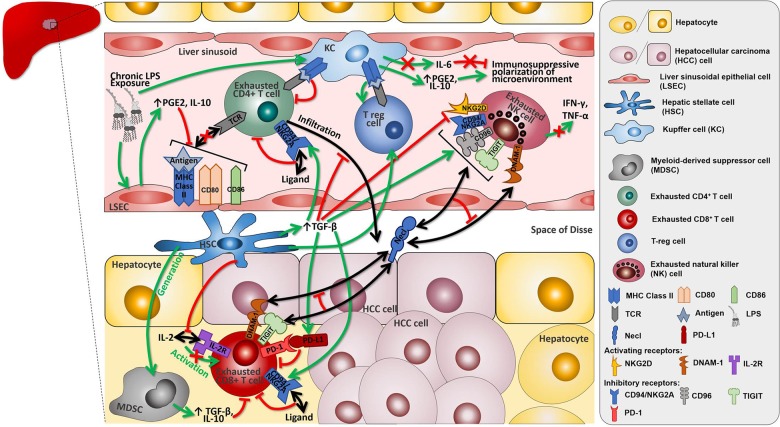
The complex and multi-faceted functional interactions guiding cancer immune tolerogenesis in hepatocellular carcinoma. Cellular and functional heterogeneity of the HCC tumour microenvironment.

Alongside LSECs and HSCs, KCs contribute to the liver immune microenvironment as non-migratory liver resident macrophages sitting at the sinusoidal interface with a high phenotypic plasticity in response to danger signals [24]. KCs respond to damage via expression of a vast repertoire of toll-like receptors, scavenger receptors as well as complement and Fc-gamma receptors [25]. In homoeostatic conditions, KC-mediated antigen presentation promotes tolerogenic immunity by attenuation of CD4+ T-cell responses, and T-reg expansion [26]. In response to LPS, KCs polarise the liver sinusoidal microenvironment towards immune-suppression by IL-6 downregulation and IL-10 release [27]. Mechanistic evidence produced to date suggests tolerance to be a key trait in liver immune homoeostasis (Fig. 1) [28].

## Immune-mediated mechanisms in the pathogenesis and progression of HCC

### T-cells

It has been long time recognised that increased T-lymphocyte infiltrate is associated with improved overall and progression free survival in HCC [29]. However, despite T-cell infiltration tumours ultimately progress and metastasise as a result of the ‘exhaustion’ of pro-inflammatory T-cell populations and accumulation of regulatory T-lymphocytes [30–32]. Cytotoxic CD8+ T-cells (CTLs) are a critical component of anti-tumour immunity in HCC and harbinger of favourable prognosis [33]. CTLs initiate T-cell receptor (TCR)-mediated, antigen-dependent cytotoxicity against tumours, being capable of directly inducing cell death via membrane-bound FAS-ligand and inhibit tumour proliferation via IFN-γ secretion [7]. CD4+ T-helper cells are an integral part of anti-tumour immunity: when activated in the presence of dendritic cell derived type-1 interferon and IL-12, they produce a number of pro-inflammatory (‘TH_1_’) cytokines, which promote CTL proliferation and thus anti-tumour immunity [34]. Overexpression of TH_1_ cytokines (IFN-γ, IL-2, IL-1α, IL-1β) is associated with favourable prognosis [35].

T-cell exhaustion is characterised by impaired pro-inflammatory responses upon stimulation, reduced cytokine production, impaired proliferation and reduced cytotoxicity. Such phenotype is hallmarked by over-expression of co-inhibitory receptors including CTLA-4, PD-1, LAG-3 and TIM-3. Evidence of intra-tumoural and circulating of exhausted CD8+ T-cells is documented as a poor prognostic trait in HCC [36, 37].

Exhaustion within the TME is multifactorial and dominated by a cytokine milieu rich in IL-10 and TGF-β that prohibits activation of CTLs and TH_1_ CD4+ T-cells [38, 39]. Single cell analysis of TCR sequences has recently confirmed clonal expansion of exhausted CD8+ T-cell clusters in HCC, indicating that CTL clones expand within the tumour after infiltration and become exhausted [40]. Novel transcriptional regulators of T-cell exhaustion are being increasingly appreciated including TOX, a transcription factor heavily overexpressed in CD8+ TILs that suppresses effector and memory function [41].

The PD-1/PD-L1 pathway is a key actionable driver of immune-exhaustion in HCC, and works by suppression of T-cell receptor signalling via the PI3K/AKT pathway, ultimately inhibiting T-cell survival and growth [42, 43]. High expression of PD-1 [36] and PD-L1 is generally associated with poor prognosis in HCC [44–46] where PD-1 over-expressing TILs can restore their effector function following PD-1 blockade [47]. Alongside PD-1/PD-L1, a growing number of inhibitory pathways has been identified to target anti-tumour CTL function.

CTLA-4 is a well described inhibitory receptor, which is upregulated after T-cell activation and is thought to act by competitively antagonising CD80 and CD86 co-stimulatory molecules and by downstream inhibition of AKT [48].

TIM-3 is expressed on CD4+, CD8+ TILs and intra-tumoural T-regs in HCC, co-localising with its ligand Galectin-9 expressed on APCs. TIM-3 expression leads to reduced CTL capacity [49–51] and higher circulating TIM-3 concentration is associated with HCC susceptibility in HBV-carriers [52].

Other drivers of T-cell exhaustion in HCC include LAG-3, associated to hypofunctional CD8+ responses in HCC TILs, which can be reversed upon LAG-3 blockade [53] and B and T-lymphocyte attenuator (BTLA), seen in >50% of PD-1+ TILs in HCC and denoting particularly pronounced hypo-functionality [54] and T-cell exclusion [55].

### Regulatory T-lymphocytes

Regulatory T-cells (T-reg) are CD4+/CD25+/FOXP3+immune-suppressive T-cells whose accumulation in HCC is associated with disease progression [56] and reduced survival [31, 57]. They are recruited intratumorally via CCL17/CCL22 secretion by tumour associated macrophages (TAMs) [58, 59]. In addition, T-reg differentiation is promoted by the production of TGF-β, IL-10 and other mediators including COX-2 and indoleamine 2,3-dioxygenase by stromal and tumour cells [60].

T-regs impair antigen presentation by down-regulating DC expression of CD80 and CD86 [61]. They can directly hamper the cytotoxic capacity of CTLs through the production of suppressive cytokines including TGF-β and IL-10 and by competing for and antagonising the effect of IL-2. T-regs can also directly lyse antigen presenting cells via granzyme-mediated cytolysis and crucially express high amounts of the inhibitory signalling molecules mentioned above [62]. Circulating T-regs correlate negatively with survival in HCC [63].

Other suppressive lymphocyte populations have been characterised in the HCC TME.

TH_2_ CD4+ T-helper cells, generated when activation occurs in the presence of IL-10 derived from intra-tumoural myeloid cells, exert CTL-inhibitory functions [64]. IL-10 is also produced by another regulatory T-cell population, Tr1 cells. These are induced in HCC by interaction with plasmacytoid dendritic cells via ligation of ICOS [65]. Increased expression of TH_2_ cytokines (IL-4, IL-5 and IL-10) is associated with disease progression and metastasis in HCC [35] as a likely consequence of IL-4-mediated recruitment of TAM, which in turn secrete TGF-β and vascular endothelial growth factor (VEGF) [35]. IL-17 producing TH_17_ subsets have also been reported in HCC. TH_17_ intratumoural density leads to poor survival through the fostering of angiogenesis [66].

Circulating TH_17_ cells in HCC can suppress autologous anti-tumour CTL responses when co-cultured in vitro [67] and are increased in more advanced disease [68].

### NK cells

NK cells are innate lymphoid cells that constitute ~30% of liver resident lymphocytes—in sharp contrast with the lower frequency of peripheral blood NK cells (range 5–15%) [69]. NK cells represent a homogeneous population identified by the abundance of specific cell surface receptors (CD56, CD16). Liver resident NK cells (lrNK) are mostly CD56^bright^ CD16^dim^ that reside in the thin-walled sinusoids along Kupffer cells, NKT and T-cells in a CCR5 and CXCR6 dependent manner [70, 71]. NK cell tolerance in steady-state liver is ensured by the presence of inhibitory receptors for self MHC-I molecules, mainly killer cell immunoglobulin-like receptors (KIR) and CD94/NKG2A [72]. Conventional circulating NK cells (CD56^dim^ CD16^bright^) are being recruited to the inflamed liver and, along lrNK cells, are activated by cytokines such as IL-2, IL-12, IL-18, IL-15 secreted by hepatocytes and Kupffer cells [73]. Upon activation, NK cells operate rapidly, without the requirement for antigen presentation, by producing cytokines (mainly IFN-γ, TNF-α), chemokines and triggering target cell apoptosis via death-inducing molecules—the FAS receptor and the TNF-Related Apoptosis Inducing Ligand (TRAIL)—and via the release of cytotoxic granules [74].

NK cell activation relies on germ-line encoded stimulatory receptors triggered by self-ligands, viral antigens or stress-induced ligands, rapidly induced in virus infected cells and tumour cells [75]. The C-type lectin-like receptor Natural killer group 2, member D (NKG2D) is a potent anti-tumour mediator expressed on NK cells, CD8+ T-cells, γδ T-cells and invariant NKT cells [76]. Human NKG2D binds to highly polymorphic ligands called MHC class-I chain-related protein A and B, unique long 16 (UL-16)-binding proteins. High level of NKG2D receptor and/or NKG2D ligands have been reported in chronic liver diseases associated with metabolic disorders [77], HCV/HBV infection [78, 79] and in HCC [80, 81].

**Fig. 2:**
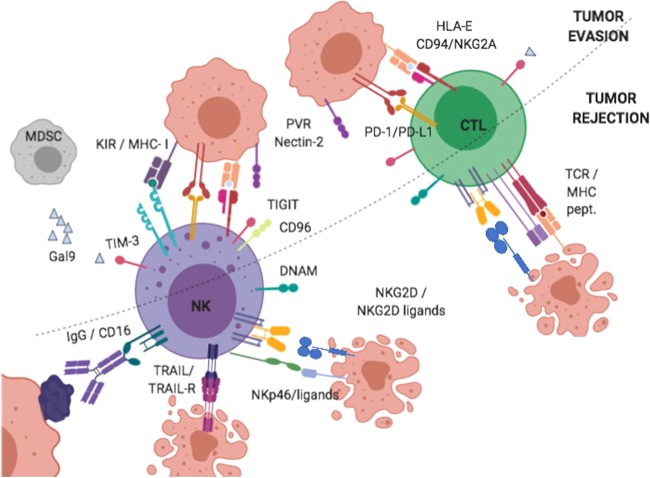
Principal functional networks driving NK cell function in hepatocellular carcinoma. Key stimulatory and inhibitory interactions involved in NK cell/tumour cell recognition and killing.

Other stimulatory receptors include the natural cytotoxic receptors (NCR) NKp46, NKp30 and NKp44; the FcɣRIII (CD16) that mediates antibody-dependent cell cytotoxicity against IgG-coated target cells, and co-stimulatory molecule such as the DNAX accessory molecule-1 (DNAM-1) triggered by adhesion molecules of the Nectin/Nectin-like (Necl) family (Fig. 2). Interestingly, Necl also interact with two inhibitory receptors CD96 on NK cells and the T-cell immunoglobulin and ITIM domain (TIGIT) on NK and CD8+ T-cells, which simultaneously prevents DNAM-1 co-stimulation, leading to reduced NK functionality [82]. Most Necl are overexpressed in HCC where high level of Nectin-4 associates with poor prognosis [83].

Dysfunctional NK cells have been described in settings of chronic inflammation such as NASH [84], viral hepatitis [85] and in the TME [86]. Reduced membrane expression of certain NKG2D ligands in HCC patients correlate with disease progression and early recurrence [87, 88]. TGF-β induces a suppressive environment in downregulating NKG2D receptors and upregulating inhibitory receptors such as TIGIT and CD96 on NK cells [89, 90] and CD94/NKG2A on NK and T-cells [91]. In HCC patients, intra-tumoral NK cells with high level of CD96 were functionally exhausted with reduced production of IFN-γ and TNFα and associated with reduced shorter disease-free survival and overall survival times [90].

### Myeloid cells

The two main myeloid cell populations within the TME are TAMs and MDSCs. MDSCs are a more immature myeloid population found in both the peripheral circulation and within tumours, whereas TAMs are tissue-resident only. Endothelial and HSC production of CXCL12 promotes intra-tumoural myeloid cell recruitment via chemokine receptor CXCR4 [92]. Infiltrating MDSCs foster tumour progression [93] and higher circulating MDSCs increase the risk of HCC recurrence after ablative therapy [94]. MDSCs deplete arginine in the TME through the production of arginase, resulting in suppression of T-cell proliferation. In addition, they promote T-reg expansion through the production of IL-10 and TGF-β and promote inhibitory signalling in effector T cells through surface expression of PD-L1 [95].

TAMs are the predominant tumour-infiltrating leucocyte population and their presence confers poor prognosis in HCC [96]. High IL-10 secretion by MDSCs results in the skewing of resident macrophages and infiltrating monocytes to an immune-regulatory phenotype. They release growth factors including TGF-β and VEGF to promote tumour growth and development, promote cancer cell stemness through activation of NF-κB and metastasis through the production of matrix metalloproteinases [97]. TAMs can also directly inhibit anti-tumour cytotoxic T-cell proliferation and promote regulatory CD4+ T-cell expansion via surface expression of PD-L1, secretion of IL-10 and TGF-β and through the production of nitric oxide and arginase in the same manner as MDSCs.

## Immune checkpoint inhibition in HCC

The systemic treatment of patients with HCC has traditionally been challenged by the intrinsic chemoresistance [98] and concomitant liver dysfunction, a competing risk factor for treatment-related toxicity and mortality [99]. The mainstay of treatment for patients who are not candidates for liver transplantation, resection or loco-regional therapies is multi-targeted molecular therapy with sorafenib [100] or lenvatinib [101], with regorafenib being an approved second-line treatment option in sorafenib-progressors [102]. Whilst effective in improving survival, these therapies are largely cytostatic and therapeutic resistance is a significant limitation to long-term survivorship.

**Fig. 3:**
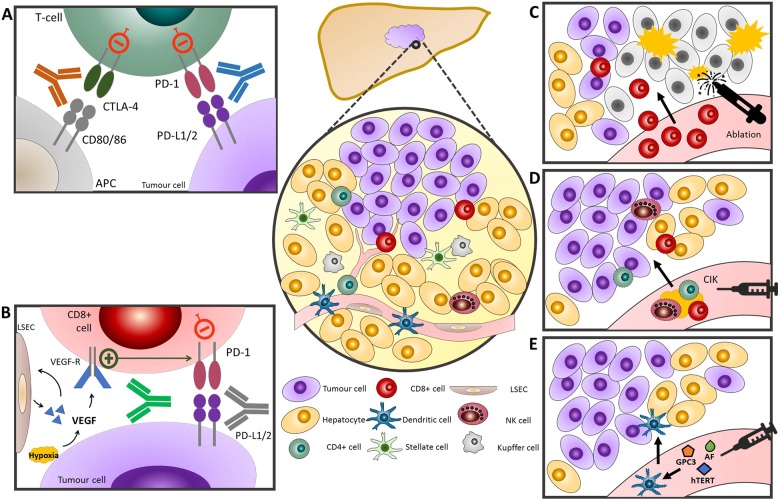
General overview of immune-based therapies for HCC. **a** Simultaneous inhibition of CTLA-4 and the PD-1 axis by monoclonal antibodies (brown and blue respectively). The effect of dual checkpoint blockade on T-cell immune reconstitution is demonstrated, with CTLA-4 acting mainly on T-reg cells and antigen-presenting cells, and PD-1 acting on effector CD8+ CTLs. **b** Schematic representation of synergy between anti-angiogenic therapy (green antibody) and PD-1/PD-L1-targeted therapy. **c** Locoregional therapies, such as ablation and trans-arterial chemoembolisation are loco-regional inducers of immunogenic cell death and drive CD8+ cell infiltration into the tumour microenvironment, providing a rationale for combined anti-PD-1 therapy. **d** Autologous T cell transfer involves ex vivo activation of mixed T cell/NK cell populations by cytokines (i.e., CIK cells) and reinfusion into the patient with the intent of bypassing immune-evasion and eliciting an anti-tumour responses. **e** Anti-tumour vaccines against immunodominant peptides of oncofoetal proteins, such as AFP, GPC3 and hTERT, have been combined with ex vivo activation of dendritic cells to promote effective antigen presentation.

Targeted blockade of CTLA-4 and PD-1/PD-L1 as forerunner molecular targets of cancer-related immune exhaustion has rapidly extended to HCC based on the promising results of ICPI therapy in multiple indications (Table 1, Fig. 3).

**Table 1:** Summary of principal active and completed clinical studies evaluating efficacy and safety of immune checkpoint inhibitors in advanced/unresectable HCC.

Clinical trial	Phase (n)	Key inclusion criteria	Drug	Efficacy data	Highest grade toxicity	NCT
	II (*n* = 21)	Hepatitis C-related HCC, Child-Pugh A or B	Tremelimumab	ORR 17.6%; mTTP 6.5 months; mOS 8.2 months; 1-year survival rate 43%	Grade 3: bilirubin elevation (10%), Grade 3–4: transaminitis (45%), rash, diarrhoea, neutropenia (5%)	NCT01008358
	I/II (*n* = 32)	BCLC B/C HCC not amenable to curative resection, RFA, or transplantation	Tremelimumab (3.5 mg/kg, 10 mg/kg)	6-month PFS 57.1%; 12-month PFS 33.1%; mTTP 7.4 months; mOS 12.3 months	Grade 3–4: AST increase (19%), ALT increase (8%), hyperbilirubinemia (8%)	NCT01853618
CheckMate-040	I/II dose escalation (*n* = 48)	Advanced HCC Virally-uninfected, HBV infected, HCV infected	Nivolumab	ORR 15%; 9-month OS rate 66%	Grade 3–4: lipase elevation (13%), AST increase (10%)	NCT02828124
	I/II dose expansion (*n* = 214)	Advanced HCC 57 uninfected sorafenib refractory, 56 uninfected sorafenib naïve/intolerant, 50 HCV, 51 HBV	Nivolumab	ORR 20% (uninfected sorafenib refractory 21%, sorafenib naïve/intolerant 23%, HCV 20%, HBV 14%); 9-month OS rate 74%	Grade 3–4: (19%) comparable to the safety profile observed in the dose-escalation phase	NCT02828124
	I/II (n = 148)	Cohort 4 Checkmate-040: Advanced HCC, Child-Pugh A class and prior sorafenib treatment	nivolumab + ipilimumab	ORR 31%; 24-mo OS rate 40%	Grade 3-4 (37%): most common: pruritus and rash 5% had grade 3-4 TRAEs leading to discontinuation	NCT01658878
CheckMate-459	III (n = 726)	Unresectable Child-Pugh A HCC naïve to systemic treatment	nivolumab vs sorafenib	OS (HR: 0.85, 95%CI: 0.72-1.02; p = 0.0752) mOS 16.4 nivolumab, 14.7 sorafenib ORR: 15% nivolumab, 7% sorafenib.	Treatment related Grade 3-4 toxicities Nivolumab (22%) Sorafenib (49%) Discontinuation rates Nivolumab (4%) Sorafenib (8%).	NCT02576509
KEYNOTE-224	II (n = 104)	Advanced HCC Child-Pugh A after sorafenib failure or intolerance	pembrolizumab	ORR:17%; 12-mo OS rate 54%; mPFS 4.9 months; mOS 12.9 months	Grade 3 (24%): transaminitis (11%), fatigue (4%) Grade 4: hyperbilirubinaemia (1%)	NCT02702414
KEYNOTE 240	III (n = 413)	Advanced HCC Child-Pugh A after sorafenib failure or intolerance	pembrolizumab vs placebo	OS (HR: 0.78; one sided p = 0.0238) and PFS (HR: 0.78; one sided p = 0.0209); ORR 16.9%	Treatment-related grade 3-4 AEs pembrolizumab (18.6%), placebo (7.5%)	NCT02702401
	Ib (n = 26)	Unresectable HCC Child-Pugh A progressed on, intolerant to or refused first-line systemic therapy	cemiplimab	partial response 19.2%, stable disease 53.8%; median PFS 3.7 months	Grade≥3 : hyponatraemia (11.5%), autoimmune hepatitis (7.7%), increased AST (7.7%) 1 death with hepatic failure considered possibly related to treatment	NCT02383212
	II (n = 220)	Unresectable HCC Child-Pugh A progressed on or intolerant to at least one line of systemic therapies	camrelizumab	ORR 13.8%; 6-mo OS rate 74.7%; median PFS 2.6 months	Grade≥3 (19.4%): any grade: increased AST (24.4%), increased ALT (23.0%), proteinuria (23.0%)	NCT02989922
	I/II (n = 40)	Advanced HCC Child-Pugh class A did not respond to or refused first-line standard therapy	durvalumab	ORR 10.0%; median OS 13.2 months; 12-mo OS rate 56.1%; 12-mo PFS rate 20.7%	Grade 3-4 (20.0%): increased AST (7.5%), increased ALT (5.0%)	NCT01693562
	I/II (n = 40)	Advanced HCC Child-Pugh A 20 uninfected, 11 HBV, 9 HCV 1st line	Tremelimumab + durvalumab vs tremelimumab	ORR 35% (uninfected), 20% (all)	Asymptomatic grade≥3 increased AST (10%), 3 pts discontinued due to asymptomatic grade 4 elevated AST and ALT, grade 3 pneumonitis, grade 3 colitis/diarrhea	NCT02519348
HIMALAYA	III (n = 1310)	Unresectable Child-Pugh A HCC naïve to systemic treatment	tremelimumab + durvalumab vs durvalumab vs sorafenib	pending	pending	NCT03298451
	Ib (n = 68)	Unresectable or metastatic HCC Child Pugh A naïve to systemic treatment (33 HBV, 22 HCV, 13 non-viral)	atezolizumab + bevacizumab	ORR 34%; 6-mo PFS 71%; median OS and median DOR have not yet been reached	Grade 3-4 (25%): hypertension (12%), Grade 3 serious AEs (7%), immune-related requiring systemic corticosteroid (6%)	NCT02715531
IMbrave150	III (n = 480)	Unresectable Child-Pugh A HCC naïve to systemic treatment	atezolizumab + bevacizumab vs sorafenib	OS (HR 0.58, 95%CI 0.42-0.79, p = 0.0006), PFS (HR 0.59, 95%CI 0.47-0.76, P<0.0001) ORR (27 vs 12%, p<0.0001)	Treatment-related Grade 3-4 atezolizumab+bevacizumab (36%), sorafenib (46%)	NCT03434379
Keynote-524	Ib (n = 30)	BCLC B/C HCC, Child-Pugh class A	pembrolizumab + lenvatinib	ORR 36.7% (RECIST) 50.0% (mRECIST)	Any grade (93%): decreased appetite (63%), hypertension (60%) 7 patients discontinued treatments due to TRAEs.	NCT03006926
VEGF Liver 100	Ib (n = 22)	1st line BCLC B/C, Child-Pugh class A, not amenable to local therapy	avelumab + axitinib	ORR 13.6% (by RECIST), 31.8% (by mRECIST); median PFS 5.5 months (by RECIST), 3.8 months (by mRECIST)	Grade 3: hypertension (50.0%), hand-foot syndrome (22.7%) no grade 4-5 TRAEs were reported. no grade≥3 irAEs were reported.	NCT03289533
COSMIC 312	III (n = 740)	1st line BCLC B/C, Child-Pugh class A, not amenable to local therapy	Cabozantinib vs Cabozantinib + atezolizumab vs Sorafenib	Pending	Pending	NCT03755791
LEAP-002	III (n = 750)	1st line BCLC B/C, Child-Pugh class A, not amenable to local therapy	Lenvatinib + pembrolizumab vs Lenvatinib	Pending	Pending	NCT03713593
	III	1st line	tislelizumab vs sorafenib	pending	pending	NCT03412773

### CTLA-4 monotherapy

Therapeutic targeting of CTLA-4 has resulted in the first demonstrable evidence of anti-tumour regression following selective modulation of a T-cell co-inhibitory pathway in melanoma [103]. Whilst characterised by low frequency of anti-tumour responses, single-agent anti-CTLA-4 therapy leads to long-term survivorship in ~20% of patients, providing evidence of long-lasting immune reconstitution [104].

Tremelimumab, a fully human anti-CTLA-4 IgG2 monoclonal antibody was the first immune checkpoint blocker to be tested in advanced HCC in a 21-patient cohort of advanced, HCV-associated HCC patients. Response rates were modest (17%), mirrored by a median time to progression (TTP) of 6.5 months [105]. Whilst lacking long-term survival data, the reported median overall survival (OS) was 8.2 months with a 43% probability of survival at 1 year. In another open-label single-arm study, Duffy et al. tested tremelimumab at 3.5 and 10 mg/kg scheduled as six 4-weekly induction infusions followed by 3-monthly maintenance. During induction, at day 36, patients underwent sub-total tumour ablation with the intent to provoke synergistic immunogenic cell death. Five out of 19 evaluable patients achieved a partial response, translating into a TTP of 7.4 months and OS of 12.3 months [106]. Both studies demonstrated evidence of anti-viral activity with falling HCV RNA load and expansion of HCV-specific T-cell responses [105]. Incidence and severity of treatment-related adverse events (TRAE) were consistent with the known mechanism of action of anti-CTLA-4 blockade in other indications. Transaminitis >Grade 2 occurred in up to 45% of patients across studies being mostly transient and not associated with overt liver functional decompensation [105]. No unexpected or dose-limiting toxicities (DLTs) were documented. Unlike melanoma, anti-CTLA-4 monotherapy has not undergone extensive testing in the context of large phase III studies leaving open questions around its efficacy across diverse etiologies of chronic liver disease and its ability to induce long-lasting anti-tumour control.

### PD-1/PD-L1 monotherapy

The clinical efficacy of PD-1/PD-L1 immune checkpoint blockade resides in its ability to augment the effector function of tumour-specific CD8+ T-cells [107], resulting in tumour rejection. The favourable therapeutic index of PD-1/PD-L1 inhibitors and reproducible efficacy has broadened opportunities for therapy in patients with previously untreatable malignancies (for instance B-RAF Wild Type melanoma) or ineligible to traditional therapies (i.e., cisplatin-ineligible urothelial cancer). In HCC, concerns over concomitant liver dysfunction and hepatotropic viral infection led to the need for confirmatory studies verifying the safety of PD-1/PD-L1 blockade in this challenging patient population.

CheckMate-040 is the landmark, multi-cohort, open label, phase I/II study that has assessed the fully human anti-PD-1 IgG4 antibody nivolumab in advanced HCC patients for safety and efficacy. The phase I component of the study assessed escalating doses of nivolumab in three concurrent cohorts of patients with virus-uninfected, HBV, HCV-infected advanced HCC starting from 0.1 mg mg/kg and utilising a standard 3 + 3 design [8]. Adequate anti-viral control was mandated in patients with HBV infection. The trial completed dose escalation to 10 mg/kg every 2 weeks with no DLTs and no hepatitis flares. TRAEs were dose-unrelated and included grade 3–4 events in 25% of patients (12/48). Nivolumab was further tested at 3 mg/kg in 214 subjects with HCV (*n* = 50), HBV infection (*n* = 51) and without viral hepatitis, further stratified in sorafenib-naïve/intolerant (*n* = 56) and sorafenib-progressors (*n* = 57). Objective response rates (ORRs) were 15 and 20% across dose-escalation and expansion cohorts and the median OS was 15 months (95% CI 9.6–20.2) in the dose-escalation group. The follow-on phase III study CheckMate-459 failed to show superiority of nivolumab over sorafenib in improving OS as a first-line therapy for unresectable HCC (HR = 0.85, 95% CI: 0.72–1.02; *p* = 0.0752) [108].

The humanised anti-PD-1 monoclonal antibody pembrolizumab has been evaluated for safety and efficacy in Keynote-224, a non-randomised phase II study of 104 patients with advanced HCC who had previously discontinued sorafenib due to progressive disease (80%) or intolerance (20%). None of the 26 HCV-positive (25%) or 22 HBV-positive patients (21%) experienced worsening or re-activation of hepatitis. TRAEs of grade 3–4 intensity were seen in 26 patients (25%), the most frequent being transaminitis in 6 (6%). The encouraging efficacy of pembrolizumab is demonstrated by the ORR achieved in 18 patients (17%), 77% of whom remained in response for over 9 months. Median PFS and OS were 4.9 months (95% CI 3.4–7.2) and 12.9 months (95% CI 9.7–15.5) and 1-year OS rate was 54% (95% CI 44–63) [9]. Keynote-240 evaluated pembrolizumab in pre-treated patients with advanced HCC in a phase III, randomised, placebo-controlled design. In total, 413 patients with Child-Pugh A, PS 0-1 advanced HCC intolerant or progressors to sorafenib were randomised 2:1 to pembrolizumab (*n* = 278) or placebo (*n* = 135), receiving treatment for up to 35 cycles or earlier interruption due to progression or unacceptable toxicity. Keynote-240 confirmed the ORR observed in other single-agent PD-1 studies (16.9%) with a median duration of response of 13.8 months (95% CI 12.7–23.6). Despite an improvement in the two co-primary endpoints of OS (HR = 0.78, 95%CI: 0.61–0.99; *p* = 0.0238) and PFS (HR = 0.78, 95%CI: 0.61–0.99; *p* = 0.0209), the study failed to meet the pre-specified threshold for statistical significance [109]. Amongst the reasons considered for the discrepancy between predicted and observed effects of pembrolizumab in this patient population is the rapidly changing landscape of systemic treatment in HCC, with the advent of novel therapies such as regorafenib and cabozantinib having changed the expected survival probability of patients with advanced HCC [110].

Other PD-1 inhibitors with less mature clinical data in HCC include tislelizumab (BGB-A317) [111], camrelizumab (SHR-1210) [112] and cemiplimab (REGN2810) [113]. Data around their safety and preliminary efficacy are available mostly from dose-expansion cohorts of early-phase clinical studies and confirm incidence and intensity of treatment-related AEs that are in keeping with the mechanism of action of the compounds. The experience with camrelizumab, tested in an open-label study of 220 patients from 13 institutions in China is perhaps the most mature, with evidence of ORR of 13.8% a median PFS of 2.6 months (95%CI 2.0–3.3) and 6-months OS rate of 74.7%. In a much smaller 26-patient cohort, cemiplimab resulted in 5 PRs and 14 SDs. Tislelizumab was launched into late-phase development with RATIONALE 301 study after identification of the recommended phase 2 dose and is currently the first study to explore non-inferiority against sorafenib in a randomised controlled phase III study in advanced HCC [114].

Clinical data on anti-PD-L1 monotherapy have been presented as part of a phase I/II study evaluating the IgG1 monoclonal antibody durvalumab in an expansion cohort of 40 HCC patients with Child-Pugh Class A, 93% of whom sorafenib experienced. The study has reported an ORR of 10%, a median OS of 13.2 months and a 56% 1-year survival rate, accompanied by a safety profile characterised by a 20% incidence of G3-4 irAEs, most commonly transaminitis in up to 7% of patients [115].

### Combination therapies

The evolving clinical experience in the use of PD-1/PD-L1 inhibitors has led to the expansion of combinations aimed at improving ORR and leading to greater survival benefit versus monotherapy. Mechanisms of synergy include simultaneous targeting of multiple co-inhibitory receptors, promotion of immunogenic cell death with local treatments or radiotherapy and re-programming of the TME with targeted anti-cancer agents. The majority of these strategies have been tested in the context of small, single-arm studies in HCC, often in absence of pre-clinical models to guide efficacy testing.

### Dual immune checkpoint blockade

Convincing clinical evidence of synergy from CTLA-4/PD-1 co-inhibition in melanoma [116], kidney cancer [117] and NSCLC [118] has led to the understanding that concurrent targeting of multiple immune checkpoints leads to an increased magnitude and depth of anti-tumour immune responses across malignancies. The rationale behind dual CTLA-4/PD-1 blockade is evident from the differential and non-redundant immune-biologic role of the two pathways within the cancer immunity cycle, where CTLA-4 is a prominent driver of immune-suppression in tumour-antigen presenting cells and T-regs, whereas PD-1/PD-L1 predominantly downregulates the effectiveness of the CD8 + CTL response.

Clinically, the safety and early efficacy of three different dosing schedules of ipilimumab and nivolumab were tested in cohort 4 of the Checkmate-040 study. Advanced HCC patients with Child-Pugh A class and prior sorafenib treatment were randomised to three arms: nivolumab 1 mg/kg + ipilimumab 3 mg/kg or nivolumab 3 mg/kg + ipilimumab 1 mg/kg every 3 weeks for four doses followed by nivolumab maintenance (240 mg flat dose every 2 weeks) until disease progression or unacceptable toxicity. A third arm evaluated nivolumab 3 mg/kg + ipilimumab 1 mg/kg every 6 weeks until discontinuation due to progression or toxicity. Overall, incidence of treatment-related AEs was 37%, most common being skin toxicity. The discontinuation rate for toxicity was low at 5%. Analysis of efficacy revealed the combination to yield a 31% ORR, which compares favourably with the previous experience of nivolumab monotherapy within the same study (14%) [119].

Durvalumab and tremelimumab have been tested in combination at the dose of 20 mg/kg and 1 mg/kg respectively every 4 weeks followed by 20 mg/kg durvalumab maintenance in a phase I/II study of 40 patients with advanced HCC. In this study 70% of the patients had received prior systemic treatment and 50% were hepatitis-uninfected. The proportion of severe treatment-related AEs was 20% and discontinuation rate was 7%. The ORR was 15%, with the entirety of confirmed responses seen in uninfected patients. Disease-control rates at 16 weeks from study commencement was 57% [120]. The encouraging results from this study have led to the inception of the HIMALAYA study, a randomised, multi-centre phase III study that will compare durvalumab and tremelimumab against durvalumab monotherapy or sorafenib as first-line therapy for HCC.

### NK cell-based therapies

To enhance NK cell activity, blockade of the inhibitory KIR receptors using the anti-KIR antibody Lirilumab (IPH2102/BMS-986015) is being evaluated in combination with nivolumab or nivolumab and ipilimumab in advanced solid tumours including HCC (NCT01714739). Safety profile of Lirilumab in monotherapy or combination showed no DLTs, with the exception of increased infusion-related reactions [121, 122].

Other inhibitory receptors shared by NK cells and T-cells including TIGIT, LAG-3, TIM-3, BTLA and NKG2A have been identified as novel checkpoint blockade [123] holding promise in combination therapy with anti-PD-1/PD-L1 or anti-CTLA-4 antibodies [88]. NKG2A blockade in animal model of HCV infection led to enhanced NK and CD8+ T-cell functions promoting HCV clearance [124]. NKG2A blockade combined with anti-PD-1/PD-L1 blockade was recently shown to enhance NK and T-cell anti-tumour response [125, 126] in murine lymphoma tumour models and in human in vitro experiments [125]. Preliminary results of a phase II trial combining monalizumab, a humanised anti-NKG2A antibody, and cetuximab, an anti-EGFR in head and neck squamous cell carcinoma patients showed a 31% objective response rate (NCT02643550). These results will likely encourage trials in other types of cancer including HCC where HLA-E ligands for NKG2A have been evidenced [125]. Successful clinical translation of these therapies to HCC requires a better understanding of the regulation and function of these pathways.

### Targeted therapies

Molecularly targeted therapies have played a pivotal role in the medical management of HCC, with sorafenib having solidly remained the first and for nearly a decade the only drug therapy to demonstrate a significant OS benefit in treatment-naïve Child-Pugh A HCC patients [100]. Pursuing combination therapy with ICPI and molecularly targeted agents is not only justified by evidence of single-agent activity but also by the complex bidirectional relationship existing between angiogenesis and immunity [127]. Resistance to anti-angiogenic therapy is in fact at least in part determined by an immune-suppressive microenvironment characterised by higher T-reg infiltration and stronger PD-L1 expression [128]. The expression of PD-L1 itself is strongly placed under the transcriptional regulation of hypoxia inducible factor 1-alpha [129]. In HCC, sorafenib therapy induces tumoural PD-L1 overexpression [130], and pre-clinical evidence in mouse models suggests this to correlate with T-reg accumulation and M2-macrophage polarisation through hypoxia, drawing a translationally appealing rationale for combination therapy [131]. Inhibition of tumour angiogenesis, and in particular VEGF aids normalisation of the endothelial barrier by regulating key adhesion molecules for immune cell homing to the tumour. VEGF also inhibits DC maturation and accentuates PD-1 expression of tumour-infiltrating CD8+ T-cells highlighting the potential for synergy between VEGF inhibition and ICPI therapy [132].

An increasing number of studies of PD-1/PD-L1 inhibitors and anti-angiogenics is underway. On the basis of the positive safety and efficacy data from GO30140 [133], the combination of atezolizumab (1200 mg) co-administered 3-weekly with bevacizumab (15 mg/kg) (A + B) was the first to reach efficacy in the phase III Imbrave 150 study (*n* = 501), the first to show superiority of combination immunotherapy in improving OS (HR 0.58, 95% CI 0.42–0.79, *p* = 0.0006), PFS (HR 0.59, 95% CI 0.47–0.76, *P* < 0.0001) and ORR (27 vs 12%, *p* < 0.0001) versus sorafenib. The favourable safety profile, with mainly asymptomatic toxicities (proteinuria/hypertension) comes as a great challenge to standard of care and is likely to change the treatment landscape of HCC [134].

Keynote-524, an open-label, phase Ib study of pembrolizumab and lenvatinib in patients with unresectable HCC echoed these results demonstrating promising anti-tumour activity and acceptable safety, although with higher rates of symptomatic toxicities. After a safety lead-in of 6 patients and an expansion of 24 patients previously untreated for HCC, at median follow up of 9.7 months ORR by RECIST criteria were 36.7% increasing to 50% when modified RECIST (mRECIST) criteria were used. On the basis of the promising initial results the protocol was amended to allow for the recruitment of 100 patients to study part 2 and led to breakthrough FDA approval of the combination on the basis of ORR [135].

The phase Ib VEGF Liver 100 study (NCT03289533) of the PD-L1 IgG1 antibody avelumab co-administered at the dose of 10 mg/kg every 2 weeks with the VEGF receptor kinase 1, 2, 3 inhibitor axitinib has demonstrated an ORR of 13.6% based on RECIST 1.1 and 31.8% when evaluated by mRECIST criteria. Median PFS was 5.5 and 3.8 months based on RECIST and mRECIST respectively. Despite fairly high levels of grade 3 TKI-related toxicities including hypertension (50%) and hand–foot syndrome (22.7%), no grade >3 irAEs and no treatment related were reported [136].

### Loco-regional therapies

Loco-regional therapies including ablation and trans-arterial chemoembolization (TACE) have traditionally played a major role in the treatment of liver-confined HCC. Mounting evidence suggests loco-regional therapies to produce quantifiable changes in immune cell subsets, leading to the premise that the local ischaemic and cytotoxic effect provoked by thermal, radio-frequency ablation or chemoembolization of liver tumours might promote immunogenic cell death [137]. The pilot study of tremelimumab combined with ablation/chemoembolisation is to date the most comprehensive prospective study that has looked at the synergistic effect between local and systemic therapy [106]. In a subset of patients who experienced clinical benefit, evidence of increased CD8+ infiltrate in tumour biopsies collected post treatment gives a positive although preliminary confirmation of the immunogenic activity of the combination. The lack of a comparator arm makes it impossible to disentangle the superadded effect of CTLA-4 inhibition from that of local therapy. Equally, the heterogeneity of ablative techniques used makes the clinical outcomes difficult to generalise to the broader population of patients with liver-confined HCC. A number of studies are actively recruiting in the intermediate-stage HCC space, with nivolumab, pembrolizumab being tested in combination with conventional (NCT03397654, NCT0314370), DEB-TACE and yttrium-90 radioembolization. Preliminary results of the PETAL clinical trial of pembrolizumab administered 3-weekly after conventional TACE has shown no evidence of synergistic toxicity with TACE [138]. Efficacy results of these early-phase clinical studies are eagerly awaited given the failure of anti-angiogenic therapy to promote clinically-significant improvements in long-term anti-tumour control and survival in this patient population [139].

## Other immunotherapeutic approaches

Beyond ICPIs, a number of other immunotherapeutic approaches have been studied over the years in primary liver cancer. The existence of measurable, naturally occurring responses against TAA in peripheral blood of patients with HCC has prompted the investigation of anti-tumour vaccine studies against immunodominant peptides of onco-foetal proteins such as alpha-fetoprotein (AFP), glypican-3 (GPC3), telomerase reverse transcriptase and many others such as MAGE-A1, NY-ESO-1 [140].

A key barrier to the effective development of vaccines as therapies for HCC stands in the co-existence of multiple inhibitory mechanisms including enrichment of infiltrating T-regs [140]. Whilst strategies including pre-conditioning with low-dose cyclophosphamide (a T-reg depleting agent) or stimulation with colony stimulating factors have been attempted [141], these have been unsuccessful in producing long-term, clinically meaningful anti-tumour responses. Ex vivo stimulation of DCs aims to correct a key primary mechanism of cancer immune evasion by promoting effective antigen presentation and induce immunological memory [142]. Results of DC-based vaccinations have however been mixed in the clinic. Whilst capable of generating measurable anti-TAA responses [143], clinical efficacy is limited to disease stabilisation with low-frequency proportion of partial responses in most studies [143–145]. Heterogeneity in DC vaccination manufacturing and the need for dedicated facilities for apheresis and re-infusion of DCs makes this approach difficult to adopt in absence of more convincing evidence of efficacy [146].

Adoptive cell transfer consists of the re-infusion of autologous T cells following in vitro activation with exogenously supplemented cytokines. Infusion of autologous T cells for 6 months following in vitro stimulation with CD3 and IL-2 has been evaluated in a randomised controlled study of 76 patients. After a median follow-up period of 4.4 years, adoptive T-cell immunotherapy decreased recurrence probability by 18% [147]. Beneficial effects from adoptive re-infusion of activated cytokine induced killer cells, a mixed population of CD3+/CD56+ and CD3+/CD56- T-cells and CD3-/CD56+ natural killer cells, were confirmed in studies enroling patients who achieved complete response following resection, ablation or percutaneous ethanol injection [148]. Combined infusion of T cells and DCs has also been attempted, leading to improvement in post-operative survival [149]. The positive results from adoptive cellular immunotherapy and the evidence of TAA-specific responses in HCC have stimulated research into chimeric antigen receptor T-cells, which combine antigen-specificity with the potential to infuse a fully active cytotoxic effector T-cell population. Whilst initial pre-clinical evidence is promising [150, 151] concerns over toxicity warrants a careful clinical development in patients with HCC [152].

## Predictive biomarkers

The probability of achieving clinical benefit from ICPI therapy is restricted to a fraction of patients with HCC. High-throughput studies on tissue samples have identified 25% of patients to harbour transcriptomic hallmarks of a pro-inflammatory response associated with features of adaptive or exhausted immunity [153]. There is an acute need to translate this knowledge into clinically available predictive correlates of response and survival to spare patients from potentially ineffective therapies characterised by the risk of life-threatening immunotoxicity.

### PD-L1 expression

Immunohistochemical detection of PD-L1 has emerged a putative predictor of response to PD-1/PD-L1-targeted checkpoint inhibitors with variable predictive ability across malignancies. Assessment of PD-L1 expression is challenged by clonal diversity of the antibodies used for detection, varying methodology for scoring of tumour and infiltrating cells and biologic heterogeneity of the sampled tissue. In HCC evidence for a predictive role for PD-L1 staining has been elusive. In Keynote-224, evaluation of PD-L1 expression by 22c3 PharmDx companion diagnostic assay was restricted to fresh or archival tissue from 52 of 104 participants. Combined tumoural and stromal PD-L1 expression was associated with higher ORR (*p* = 0.021) and PFS (*p* = 0.026) to pembrolizumab, whereas tumour cell staining alone was not (*p* = 0.088 and 0.096, respectively) [9]. In Checkmate 040, PD-L1 expression in tumour cells was evaluated using the 28-8 PharmDx assay. In the expansion cohort (*n* = 174), 9/34 patients with PD-L1 >1% achieved an objective response (26%) compared with 26 out of 140 (19%) with PD-L1 < 1% [8]. Analytical heterogeneity across PD-L1 assays is substantial [154], a factor that might contribute to the poor performance of this biomarker.

### Tumour mutational burden (TMB)

Tumour mutational burden (TMB) measures the number of somatic non-synonymous mutations per mega-base (Mut/Mb) in the coding genome of tumour cell [155]. It is believed that TMB-high tumours, mostly defined as those harbouring >10 mut/Mb, are enriched in tumour neoantigens and therefore intrinsically immunogenic. TMB is an emerging predictor of response across malignancies. However, compared with other tumours, HCC has median number of 5 Mut/Mb [156–159], ranging from 0.5 to 10 Mut/Mb [156, 157]. Impairment of mismatch repair mechanisms, which contribute to a hypermutated phenotype is also infrequent in HCC [160]. Analyses of genomic databases including a small fraction of ICPI recipients (*n* = 17) confirmed the low prevalence of TMB-high HCC (median 4 Mut/Mb) and limited evidence for a predictive role of TMB, highlighting the need for more comprehensive evaluation of genomic and non-genomic predictors of outcome [161].

### The gut microbiota as a source of biomarkers

Microbial proteins are able to prime T-cell responses [162] and play a fundamental role in the induction, training and function of the host immune system [163, 164]. The gut microbiome determines resistance to ICPI in a number of tumours treated with PD-1 blockade [165–167]. The liver does not harbour a known intrinsic microbiome but the gut microbiota plays a critical role in liver inflammation, chronic fibrosis, liver cirrhosis and HCC development via the gut–liver axis [168–170]. A pilot study of 8 HCC patients treated with PD-1 inhibitors has revealed taxonomic diversity and enrichment in 20 species including *Akkermansia* and *Ruminococcaceae* to predict for response [171] suggesting characteristic alterations in the gut microbiota profile to have potential as predictive biomarkers of clinical benefit in ICPI-recipients. The precise molecular mechanisms that may facilitate anti-tumour immunity warrant further elucidation in larger cohorts.

## Conclusions and future directions

With its robust immune/inflammatory pathogenesis, HCC remains a strong candidate for the development of immune-based therapies. The rapid expansion of immunotherapy has however been only partially successful. To date, cell-based therapies remain promising but limited in widespread clinical application. ICPIs yield objective anti-tumour responses but their effect in improving survival is still unproven. The plethora of redundant immune-suppressive signals coupled with the lack of robust biomarkers for stratification appear key barriers to an effective deployment of immunotherapy. Rationale selection of immunotherapeutic combinations may yield a window of opportunity to improve outcomes across the various stages of HCC.
